# Replaceability of Schiff base proton donors in light-driven proton pump rhodopsins

**DOI:** 10.1016/j.jbc.2021.101013

**Published:** 2021-07-28

**Authors:** Syogo Sasaki, Jun Tamogami, Koki Nishiya, Makoto Demura, Takashi Kikukawa

**Affiliations:** 1 Graduate School of Life Science, Hokkaido University, Sapporo, Japan; 2 College of Pharmaceutical Sciences, Matsuyama University, Matsuyama, Ehime, Japan; 3 Faculty of Advanced Life Science, Hokkaido University, Sapporo, Japan

**Keywords:** bioenergetics, membrane transport, photobiology, rhodopsin, proton transport, proton pump, ion pump, retinal proteins, BR, bacteriorhodopsin, CP, cytoplasmic, DR, deltarhodopsin, EC, extracellular, ESR, *Exiguobacterium sibiricum* rhodopsin, PR, proteorhodopsin, SB, Schiff base

## Abstract

Many H^+^-pump rhodopsins conserve “H^+^ donor” residues in cytoplasmic (CP) half channels to quickly transport H^+^ from the CP medium to Schiff bases at the center of these proteins. For conventional H^+^ pumps, the donors are conserved as Asp or Glu but are replaced by Lys in the minority, such as *Exiguobacterium sibiricum* rhodopsin (ESR). In dark states, carboxyl donors are protonated, whereas the Lys donor is deprotonated. As a result, carboxyl donors first donate H^+^ to the Schiff bases and then capture the other H^+^ from the medium, whereas the Lys donor first captures H^+^ from the medium and then donates it to the Schiff base. Thus, carboxyl and Lys-type H^+^ pumps seem to have different mechanisms, which are probably optimized for their respective H^+^-transfer reactions. Here, we examined these differences *via* replacement of donor residues. For Asp-type deltarhodopsin (DR), the embedded Lys residue distorted the protein conformation and did not act as the H^+^ donor. In contrast, for Glu-type proteorhodopsin (PR) and ESR, the embedded residues functioned well as H^+^ donors. These differences were further examined by focusing on the activation volumes during the H^+^-transfer reactions. The results revealed essential differences between archaeal H^+^ pump (DR) and eubacterial H^+^ pumps PR and ESR. Archaeal DR requires significant hydration of the CP channel for the H^+^-transfer reactions; however, eubacterial PR and ESR require the swing-like motion of the donor residue rather than hydration. Given this common mechanism, donor residues might be replaceable between eubacterial PR and ESR.

Ion-pump proteins act as energy transducers within cell membranes. They convert the energy of ATP hydrolysis or sunlight into electrochemical gradients of substrate ions across membranes. The resultant electrochemical gradients drive various metabolic processes and thus contribute to cellular homeostasis. Similar to other membrane proteins, the transmembrane regions of ion pumps consist of many hydrophobic residues. The resultant hydrophobic regions inside the proteins prevent unfavorable leakage of ions. However, these regions also make it difficult to accomplish ion transport tasks because once activated, ion pumps need to transport their substrate ions through hydrophobic regions. Thus, pumps seem to have some mechanism to overcome these difficulties. In this study, we analyzed such mechanisms of light-driven H^+^ pump rhodopsins by focusing on the differences among their subgroups.

The H^+^ pump rhodopsins belong to the microbial rhodopsin family, which is a huge family of photoactive membrane proteins widespread in unicellular microorganisms (1, 2, 3). They commonly consist of seven transmembrane helices, and the retinal chromophore binds to a conserved Lys residue *via* Schiff base (SB) linkage. Upon light absorption, the retinal undergoes isomerization from the all-*trans* to the 13-*cis* state, which in turn activates the protein by distorting the conformation. This activated state returns to the original state *via* various structural intermediates. During this cyclic reaction, called a photocycle, microbial rhodopsins exert various functions that are largely categorized into ion transporters and light sensors.

The first microbial rhodopsin identified was an H^+^ pump named bacteriorhodopsin (BR) from a highly halophilic archaeon (4, 5). Approximately 30 years after this discovery, microbial rhodopsins were still found only in haloarchaea. In 1999, however, various rhodopsins, including novel H^+^ pumps, started to be discovered in many microorganisms (6). At present, the H^+^ pump rhodopsin defines the largest functional class of microbial rhodopsins. The representative is proteorhodopsin (PR, also called green proteorhodopsin), whose relatives were identified in marine bacteria inhabiting oceans worldwide (7, 8). Thus, the PR group is the most abundant H^+^ pump rhodopsin in nature. As shown in Fig. S1, BR and PR belong to distinct phylogenetic classes and have relatively low amino acid identities (identity, 22.9%; similarity, 36.0%). Conversely, they share key amino acid residues for H^+^ transport and show almost the same photocycles. Thus, the same “H^+^ pump” task probably forces them to share machinery and to arrange the same residues at appropriate positions. However, this view was partially disrupted by an H^+^ pump from the eubacterium, *Exiguobacterium sibiricum* (ESR) (9). As described later, ESR conserves different residues from BR and PR at key positions, implying that the same task is achieved in ESR with unique machinery.

Upon illumination, H^+^ pump rhodopsins cause multiple H^+^-transfer reactions and finally accomplish H^+^ translocation across the cell membrane. Figure 1 shows these H^+^ transfer steps for BR, PR, and ESR (*lower panels*) and their respective photocycling schemes (*upper panels*) (10, 11, 12, 13, 14, 15, 16). In all H^+^ pumps, the first H^+^-transfer reactions occur during the formation of the M intermediate, where the H^+^ of the protonated SB moves to the nearby H^+^ “acceptor” Asp residues (Fig. 1, *lower panels*). For BR, this primary reaction evokes subsequent H^+^ release to the extracellular (EC) medium from the “H^+^ release group (PRG)” (Fig. 1*A*, *lower panel*). In PR and ESR, H^+^ release does not occur at this step because of the lack of PRG (Fig. 1, *B* and *C*). Instead, they release H^+^ from the acceptor residue at the latter step (N decay). Thus, PRG is not essential for H^+^ pump function, and there are no important differences in the reactions at the EC side. In contrast, the reactions at the cytoplasmic (CP) side are distinct because of the difference in H^+^ “donor” residues (Fig. 1, *lower panels*). Compared with the EC channels, their CP channels are highly hydrophobic. Thus, H^+^ pumps have special mechanisms to facilitate H^+^-transfer reactions, where the “donor” residues play central roles.

**Figure 1:**
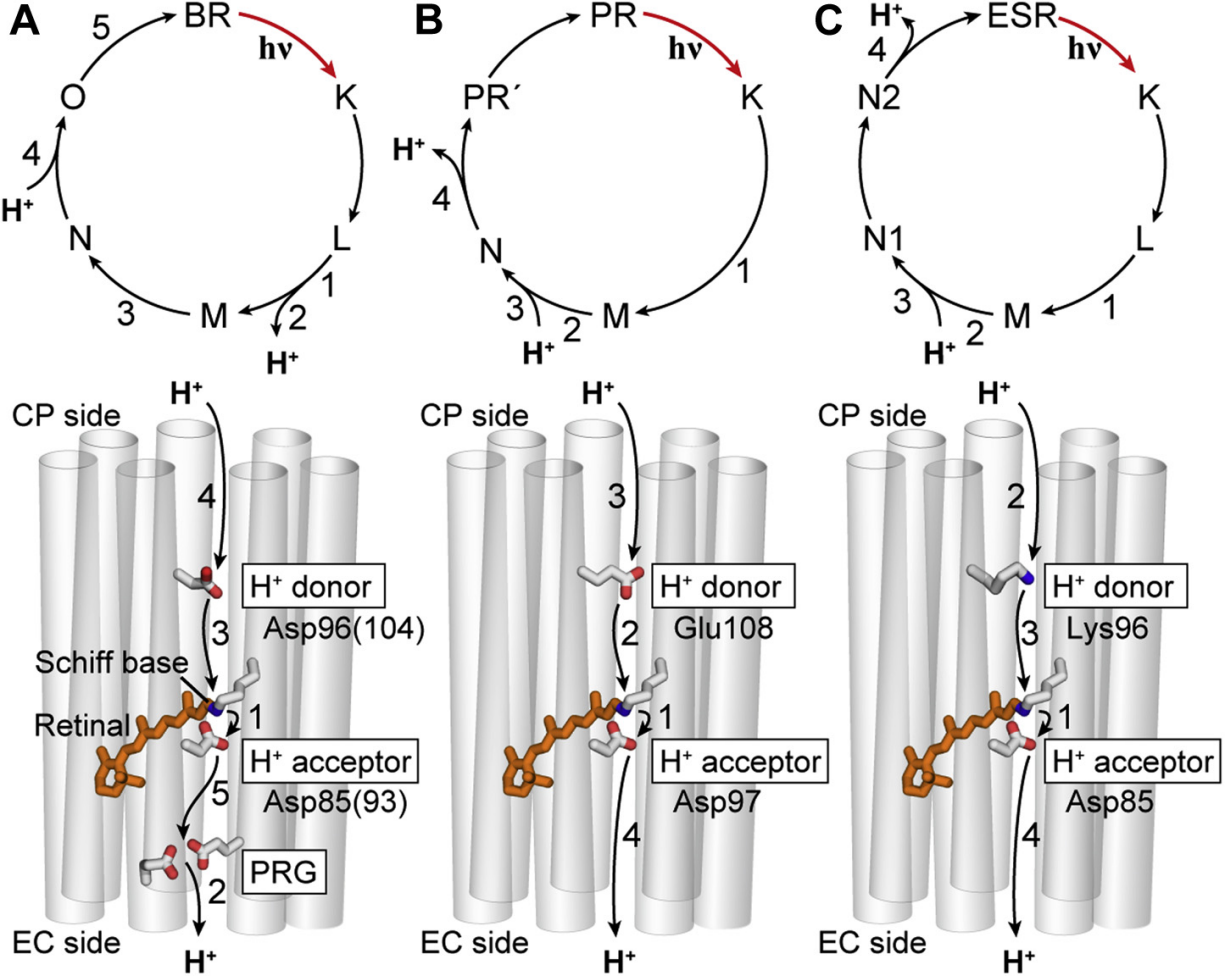
**Photocycles of BR, PR, and ESR.** Typical photocycling schemes at physiological pH and the proton transfer steps inside the proteins for BR (*A*), PR (*B*), and ESR (*C*) are shown in the *upper* and *lower panels*, respectively. Numbers 1 to 5 represent the sequences of proton transfers. The photocycle of BR is essentially the same as that of DR (17). Asp85 and Asp96 of BR correspond to Asp93 and Asp104 of DR, respectively. These residue numbers are indicated in *parentheses* in the illustration for BR. BR, bacteriorhodopsin; DR, deltarhodopsin; ESR, *Exiguobacterium sibiricum* rhodopsin; PR, proteorhodopsin.

For all H^+^ pumps, the reprotonation of the SB occurs during M decay. This H^+^ is provided by the donor residue located approximately halfway within the CP channel. In BR and PR, Asp and Glu residues act as H^+^ donors (Fig. 1, *A* and *B*, *lower panels*) (10, 11, 12, 13). In the dark state, these carboxyl residues are commonly protonated, reflecting the hydrophobic environment. Conformational changes in the photolyzed states induce H^+^ transfer from carboxyl donors to SBs. After this H^+^ donation, these donors switch their accessibility from the SB to the CP medium. Then, they capture new H^+^ from the medium. As described previously, BR and PR are phylogenetically distinct but commonly utilize carboxyl residues. Thus, carboxylates were believed to be the only residues that can act as H^+^ donors. However, this view was altered by ESR, which was revealed to use the Lys residue as the H^+^ donor (Fig. 1*C*, *lower panel*) (9, 14). Similar to carboxyl donors, the Lys residue has no charge in the dark state. As a result, H^+^ transfer occurs in the reverse order: In the ESR, the Lys residue first captures H^+^ from the CP medium and then provides it to the SB (Fig. 1*C*, *lower panel*). Despite the different H^+^-transfer reactions, the carboxyl and Lys donors greatly accelerate M decay. Thus, these donors are commonly essential for efficient H^+^ pump function. The different H^+^-transfer reactions probably require different conformational changes. Thus, carboxylate-type and Lys-type H^+^ pumps seem to have different mechanisms, which are probably optimized for their respective H^+^-transfer reactions.

In this study, we probed these differences by analyzing the replacement effects of donor residues. For the Asp-type H^+^ pump, we used deltarhodopsin (DR) instead of BR because DR can be functionally expressed in *Escherichia coli* despite its high similarity with BR (identity, 52.0%; similarity, 64.0%) (Fig. S1) (17). For all H^+^ pumps, M decay is closely related to the functionalities of the donor residues. Thus, we mainly examined the M decay rates of the donor-replacement mutants. For Asp-type DR, we found that the embedded Lys residue distorted the protein conformation and did not function as a donor residue. This result seemed to reflect a different architecture between carboxyl-type and Lys-type H^+^ pumps. However, other results were unexpected. For Glu-type PR, the embedded Lys residue functioned well. Furthermore, for Lys-type ESR, the embedded Asp and Glu residues also functioned well. Thus, PR seems to share a common mechanism with ESR. The M decays were further analyzed by focusing on the magnitudes of conformational changes associated with H^+^-transfer reactions. The results suggested that PR and ESR function in a similar mode, which requires lower hydration of the CP channel compared with that of DR.

## Results

### Overview of the photocycles of donor-replacement mutants

When the donor residue does not work, M decay becomes very slow because SB needs to capture H^+^ from the medium passing through the hydrophobic channels. Thus, we replaced the donor residues and then examined their effects on M decay. Hereafter, we used the terms “donor-replacement mutants” and “donor-disabled mutants.” The former denotes the mutants whose donors were replaced by Lys or carboxyl residues, whereas the latter mutants have nondissociable residues at the donor positions.

**Figure 2:**
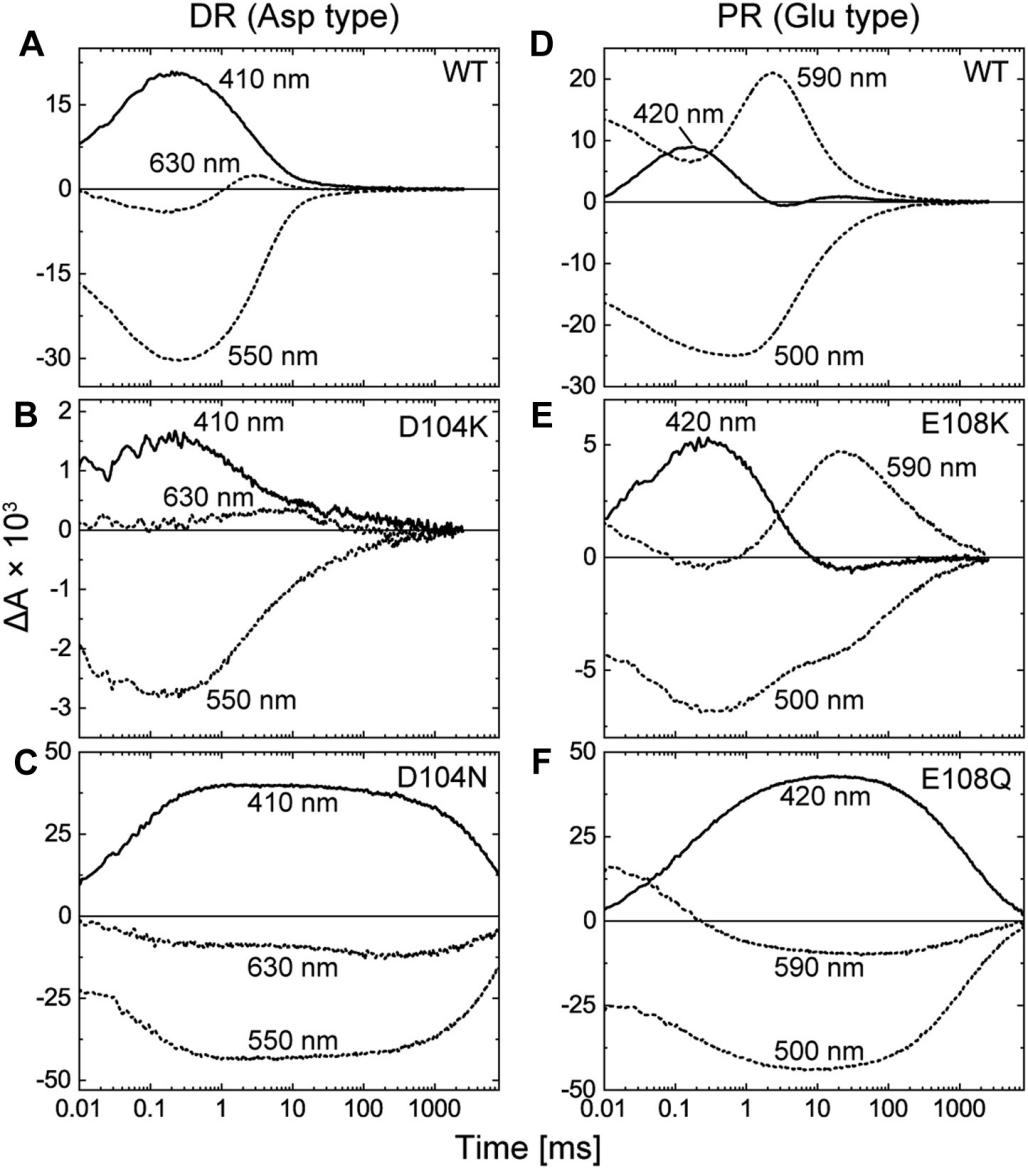
**Flash-induced absorbance changes of DR, PR, and their mutants at three typical wavelengths.** The *top panels* represent the data for WT DR (*A*) and PR (*D*), and the data for their donor-replacement mutants and the donor-disabled mutants are shown in the *middle* (*B* and *E*) and *bottom panels* (*C* and *F*), respectively. The traces at 410 nm for DR (*A–C*) and 420 nm for PR (*D–F*) reflect the formations and decays of M intermediates. These traces are shown in *solid lines*, and the others are shown in *dotted lines*. Except for donor-disabled mutants (*C* and *F*), the M decays lead the absorbance increases at 630 nm for DR and 590 nm for PR, reflecting the formations of subsequent intermediates. For PR and the mutants (*D–F*), the traces at 590 nm start with positive values (at 0.01 ms) because of the presence of remaining K. The negative deflections at 550 nm for DR (*A–C*) and 500 nm for PR (*D–F*) represent the depletions of original dark states. The pH values were 6 for DR and 8 for PR. All buffer solutions contained 0.4 M NaCl. DR, deltarhodopsin; PR, proteorhodopsin.

Figure 2 shows the results for DR and PR. Here, the photocycles were monitored by the flash-induced absorbance changes at three typical wavelengths. Each panel contains the time traces within 0.01 to 8000 ms after the flash excitation. These data reflect the photocycles after L formation for DR and after K formation for PR, respectively. The top panels (Fig. 2, *A* and *D*) indicate the results for the wildtype proteins. In addition to the wildtype proteins, all prepared samples had λ_max_ at approximately 520 to 550 nm, as shown in Fig. S2. Thus, at these wavelengths, negative deflections were observed after flash excitation, reflecting depressions of the original dark states. These signals correspond to the traces at 550 nm for DR and 500 nm for PR. Conversely, the M intermediates exhibit blueshifted absorption spectra because of the deprotonation of SB. Their formation and decay were detected at 410 nm for DR and 420 nm for PR. As shown here, the M decayed within 10 ms, which was significantly faster than the decay of donor-disabled mutants (DR-D104N and PR-E108Q), as plotted in the *bottom panels* (Fig. 2, *C* and *F*). The *middle panels* (Fig. 2, *B* and *E*) show the results for the Lys mutants (DR-D104K and PR-E108K). The replacements by Lys slowed down M decay to some extent. However, the decay rates were still significantly faster than those of the donor-disabled mutants. Thus, the embedded Lys residues appeared to function in DR and PR, even though the H^+^-transfer reactions should occur in the inverted order from the original reactions. For the donor-disabled mutants (*bottom panels*), other intermediates after M were not detected until the ends of the traces at 8 s because of the slow decay of M. However, for both the wildtype proteins and the donor-replacement mutants, the subsequent intermediates commonly appeared at longer wavelengths, reflecting the fast decay of M. For PR (Fig. 2*D*), the intermediate at 590 nm is assigned to N (12), which is the next intermediate of M and still contains 13-*cis* retinal. For DR (Fig. 2*A*), N was short lived and did not significantly accumulate. Instead of N, the next intermediate O appears at 630 nm for DR as a result of reisomerization of the retinal to the all-*trans* state. O is the last intermediate, and its decay corresponds to the recovery to the original state. For PR, N transforms to PR', which is a precursor of the original state and contains all-*trans* retinal (12, 13). Thus, the final transition (PR' → PR) involves only a faint change in the absorption spectrum.

**Figure 3:**
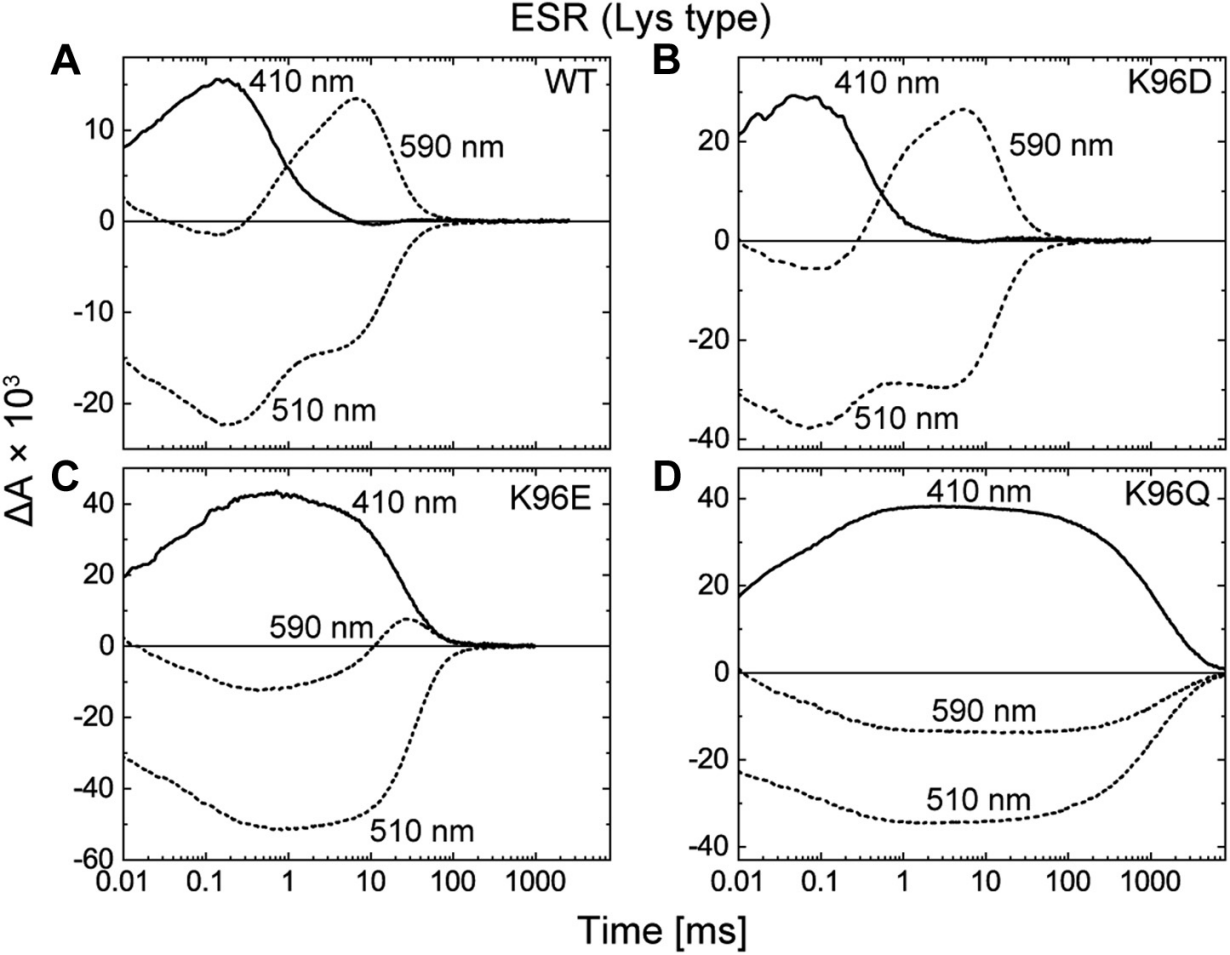
**Flash-induced absorbance changes of ESR and the mutants at three typical wavelengths.** The ESR data corresponding to those in Figure 2 are shown here. The panels *A–D* represent the data for WT ESR and the K96D, K96E, and K96Q mutants, respectively. The M decays lead to the formations of subsequent intermediates at 590 nm except for ESR-K96Q (*D*). ESR, *Exiguobacterium sibiricum* rhodopsin.

The donor-replacement effects for ESR are summarized in Figure 3, where the M intermediates were monitored at 410 nm. The time range (0.01–8000 ms) is the same as in Figure 2, and the traces reflect the photocycles after L formations. For wildtype ESR (Fig. 3*A*), M decay was completed within 10 ms. This fast decay was still observed after donor replacement by the Asp residue (K96D; Fig. 3*B*). Reflecting the fast M decay, the next intermediate appeared at 590 nm with almost the same kinetics as that of wildtype ESR. This intermediate was previously assigned to N for the wildtype protein because of the 13-*cis* configuration of retinal (14). The replacement by the Glu residue (K96E; Fig. 3*C*) slowed the M decay approximately 10-fold but was still significantly faster than that of the donor-disabled mutant (K96Q; Fig. 3*D*). Thus, both carboxyl residues seemed to function as H^+^ donors, although they probably mediate different H^+^-transfer reactions from the wildtype ESR.

### Donor-replacement effects on H^+^-pump activities

**Figure 4:**
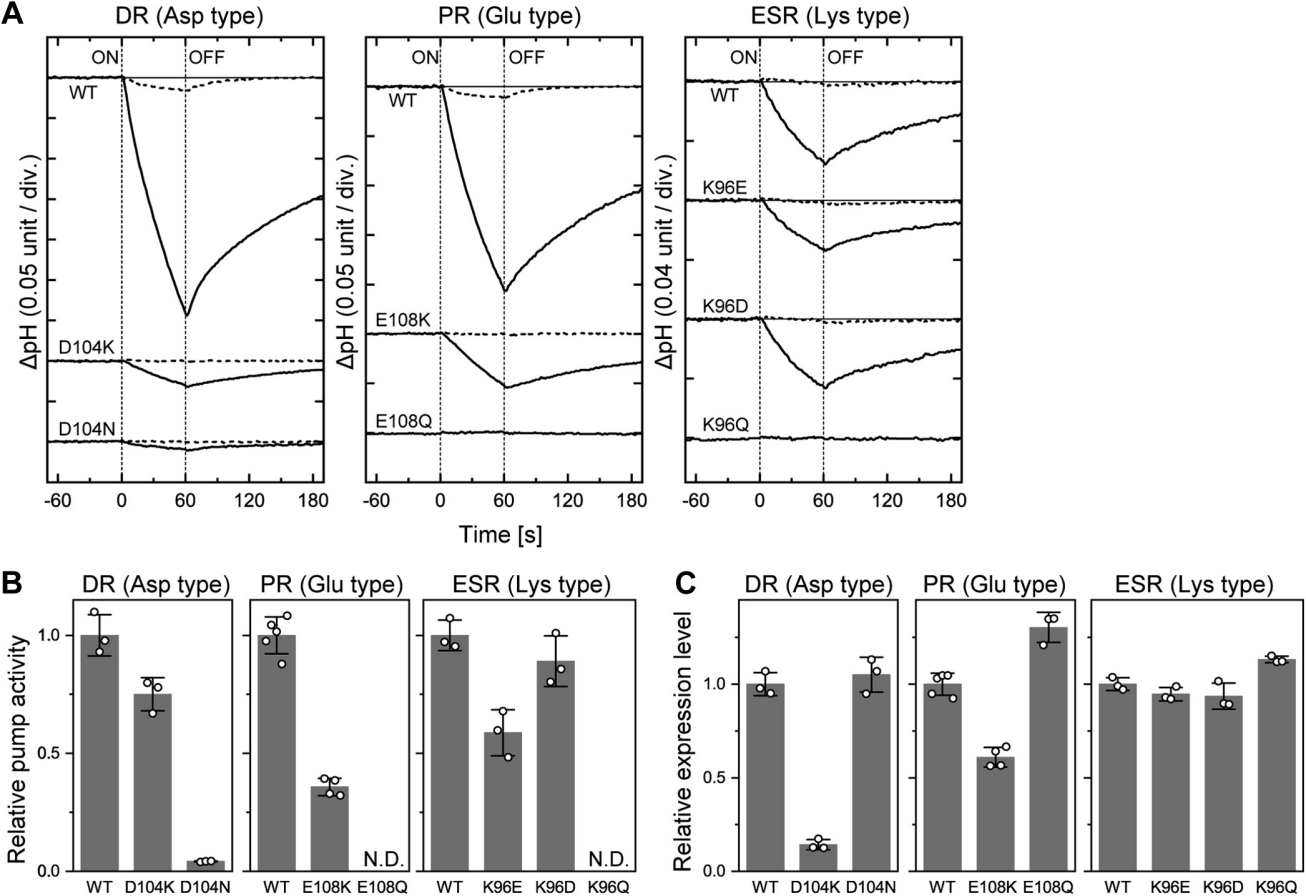
**H**^**+**^**-pump activities of DR, PR, ESR, and their mutants.***A*, time courses of light-induced pH changes of the *Escherichia coli* suspensions. Light was turned on and off at 0 and 60 s, respectively. The pH changes after additions of CCCP (10 μM) are shown with *broken lines*. The initial pH values were 5.9 to 6.3. *B*, the relative H^+^-pump activities. They were calculated by dividing the initial slopes of pH changes by their respective expression levels. *C*, the relative expression levels in the *E. coli* membranes. They were estimated from the maximum values of the flash-induced absorbance changes at λ_max_ in the dark state. In (*B*) and (*C*), the scatter of *open circles* represents each measurement, and the bar represents the mean ± standard deviation (n = 3–5). CCCP, carbonyl cyanide *m*-chlorophenylhydrazone; DR, deltarhodopsin; ESR, *Exiguobacterium sibiricum* rhodopsin; ND, not detected; PR, proteorhodopsin.

For the donor-disabled mutants (DR-D104N, PR-E108Q, and ESR-K96Q), M decay is the rate-limiting step of the photocycles (Figs. 2, *C* and *F* and 3*D*). Thus, slow M decays significantly lower the photocycling rates, which should result in faint H^+^-pump activity under constant illumination. Conversely, we observed much faster photocycling rates for the donor-replacement mutants (DR-D104K, PR-E108K, ESR-K96D, and ESR-K96E) (Figs. 2, *B* and *E* and 3, *B* and *C*). Thus, they should exhibit distinct H^+^-pump activities. Figure 4 summarizes these activities probed by the light-induced pH change of the *E. coli* suspensions. The outward H^+^ transport should decrease the pH values. These time traces are shown in Figure 4*A*, where the donor-disabled mutants exhibited only negligible pH changes (*the bottom traces*). On the other hand, distinct pH decreases were observed for not only the wildtype proteins but also the donor-replacement mutants. These pH changes disappeared after the addition of the protonophore carbonyl cyanide *m*-chlorophenylhydrazone (*broken lines*), indicating that H^+^ was indeed pumped under illumination. To evaluate the relative pump activities, the initial slopes of the pH changes are plotted in Figure 4*B* after dividing by the relative amounts of the expressed proteins, which were evaluated by the flash-induced signals summarized in Figure 4*C* (see Experimental procedures section for the details). As shown in Figure 4*B*, the donor-replacement mutants (DR-D104K, PR-E108K, ESR-K96E, and ESR-K96D) exerted significant H^+^-pump activities similar to their wildtype proteins.

### pH dependence of M decay

The results thus far suggested that the embedded Lys and carboxyl residues actually function as H^+^ donors. However, fast M decay can occur without the donor residue if the mutation largely distorts the protein structure. Indeed, the BR triple mutant D96G/F171C/F219L, in which the donor (D96) is replaced by a Gly residue, showed fast M decay because of the open structure of the CP channel even in the dark state (18). The open structure probably induces hydration of the channel and facilitates H^+^ inflow. Thus, the “true” functionality of the donor residue should be confirmed by the pH dependence of the M decay rate. If the residue does not act as an H^+^ donor, SB needs to directly capture H^+^ from the medium. As a result, the M decay rate monotonically decreases as the pH increases. Conversely, if the residue provides H^+^ to SB, the M decay rate is pH independent in a certain pH range.

**Figure 5:**
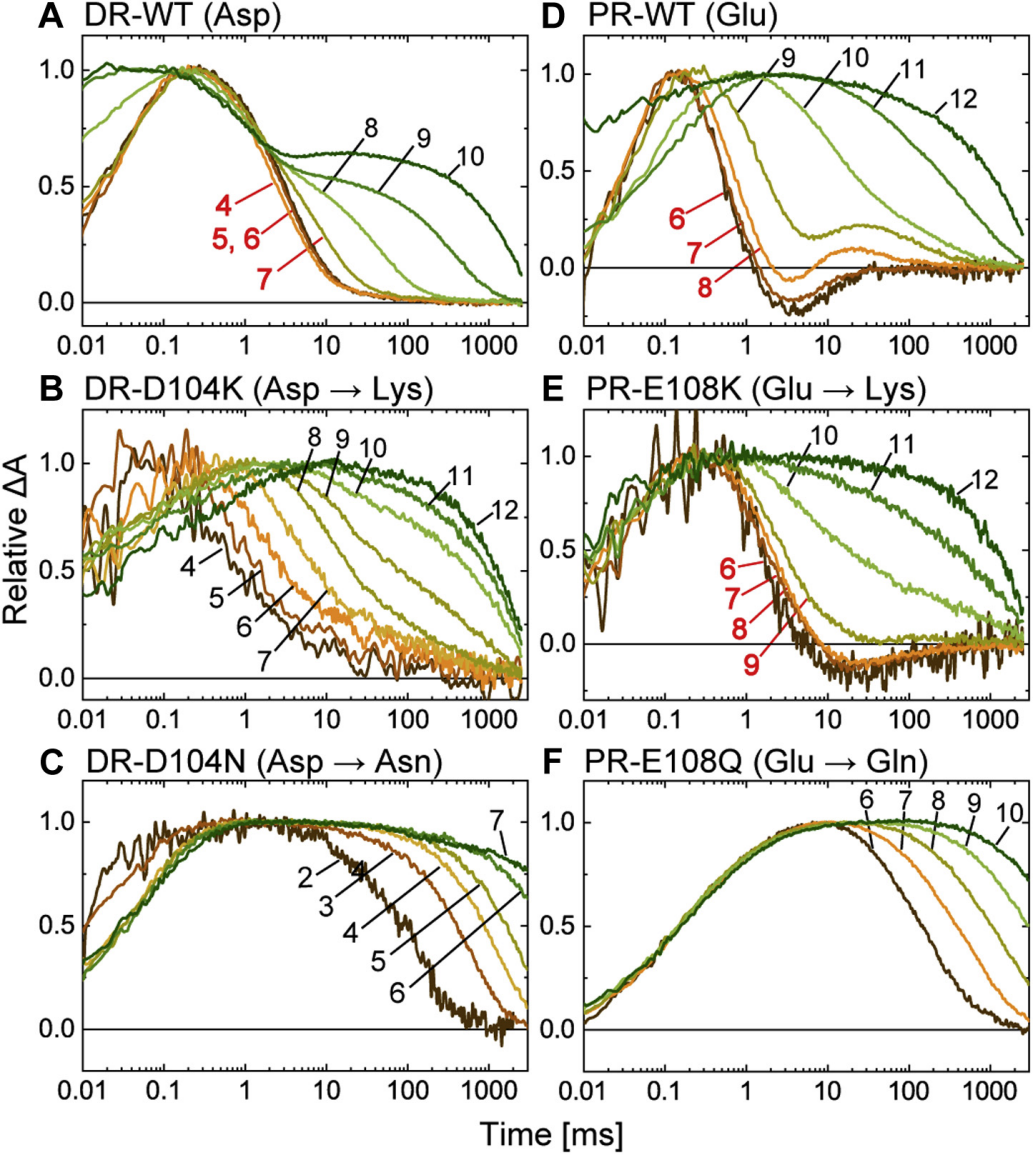
**pH dependence of M decay of DR, PR, and their mutants.** The *top panels* represent the data for WT DR (*A*) and PR (*D*), and the data for their donor-replacement mutants and the donor-disabled mutants are shown in the middle (*B* and *E*) and bottom panels (*C* and *F*), respectively. Flash-induced absorbance changes at 410 nm for DR (*A–C*) and 420 nm for PR (*D–F*) were measured at various pH values and plotted after normalization to the respective peak magnitudes. The pH values are also indicated in the panels. In panels *A*, *D*, and *E*, the M decay showed negligible pH dependence in a particular pH range. The pH values in those ranges are shown in *red*. The buffer solutions contained 0.4 M NaCl. DR, deltarhodopsin; PR, proteorhodopsin.

**Figure 6:**
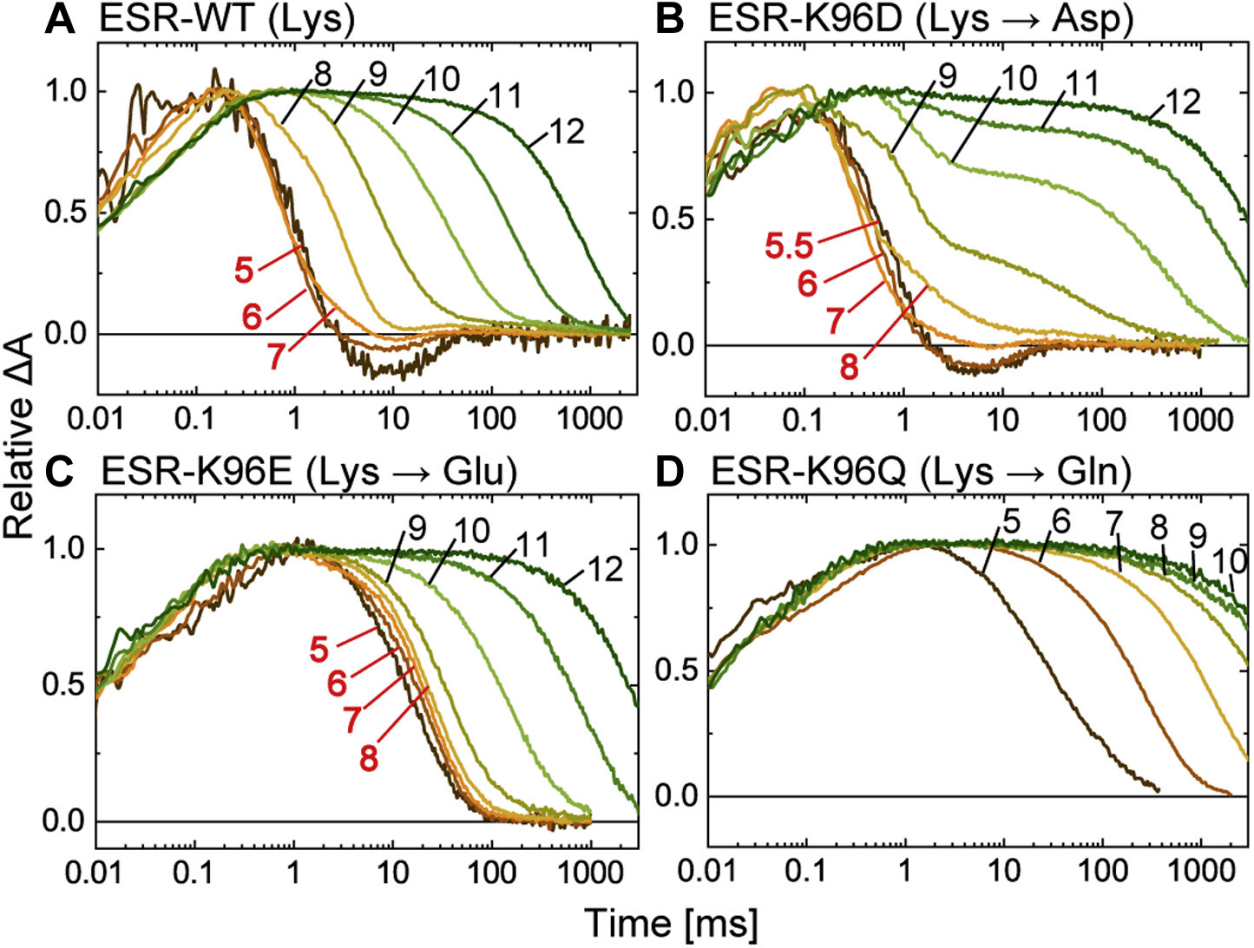
**pH dependence of M decay of ESR and the mutants.** The ESR data corresponding to those in Figure 5 are shown here. The *panels A–D* represent the data for WT ESR, and the K96D, K96E, K96Q mutants, respectively. The absorbance changes were measured at 410 nm. The pH values are shown in *red* to indicate the pH ranges where the M decay showed negligible pH dependence. The buffer solutions contained 0.4 M NaCl. ESR, *Exiguobacterium sibiricum* rhodopsin.

Figures 5 and 6 show the pH dependences of M decay, where the flash-induced absorbance changes at 410 or 420 nm are plotted. The respective pH values are indicated in the panels. For DR and the mutants, the traces above pH 4 are plotted. The lowest pH values are 6 for PR (Fig. 5, *right panels*) and 5 or 5.5 for ESR (Fig. 6) because of the p*K*as of the acceptor residues. At even lower pH values, the acceptors were completely protonated, so the yields of M were negligible. For wildtype DR (Fig. 5*A*), the M decay rates were almost constant below pH 7, whereas above pH 8, the traces showed biexponential decays. The first decay rates were almost the same as those below pH 8, indicating that the donor (D104) still provided H^+^ to the SB at any pH. On the other hand, the second decay rates became slower as the pH increased. Essentially, the same pH dependences were also observed for BR (19). The Asp donor of BR is known to have a p*K*a of 7∼8 for H^+^ uptake during subsequent N decay (20, 21, 22, 23). Thus, the N decay becomes slow with increasing pH beyond the p*K*a. As a result, N accumulates and reaches equilibrium with the preceding M. Thus, in Figure 5*A*, the first decay corresponds to the formation of MN equilibrium, and the decay corresponds to the second decay. Consequently, the Asp donor ideally functions below pH 7, where the donor can quickly perform two H^+^-transfer reactions, that is, H^+^ donation to SB and H^+^ uptake from the medium. Conversely, for DR-D104K (Fig. 5*B*), the M decay showed significant pH dependence at all measured pH values. This behavior is similar to that of the donor-disabled mutant (D104N; Fig. 5*C*), where SB directly captures H^+^ from the medium. Thus, the Lys residue does not function as a donor residue.

In contrast to DR, the embedded Lys residue surely functions in PR. The PR-E108K mutant (Fig. 5*E*) exhibited an almost constant decay rate of M below pH 9. Thus, the Lys residue acts as an H^+^ donor at almost the same pH range as that of wildtype PR (<pH 8; Fig. 5*D*). Above the respective pH ranges (pH > 8 for wildtype PR; pH > 9 for PR-E108K), M decay commonly slowed down as the pH increased, as in the donor-disabled mutant (PR-E108Q; Fig. 5*F*). Thus, both donors are disabled at high pH, but the mechanism should be different between them. The M decay in wildtype PR did not show the biexponential decay that appeared in the wildtype DR (Fig. 5*A*). Thus, above pH 8, the Glu donor is probably deprotonated even in the dark states and thus does not contribute to M decay. On the other hand, the Lys donor in PR-E108K should be deprotonated at any pH in the unphotolyzed state. Thus, the dysfunction above pH 9 might reflect the fact that the Lys donor has a p*K*a of ∼9 for H^+^ uptake. Because of this p*K*a value, H^+^ uptake slows down above pH 9 and becomes the rate-limiting step for M decay.

Similar to PR, the embedded carboxyl residues also function in ESR. For wildtype ESR (Fig. 6*A*), the Lys donor ideally functions below pH 7, up to which point the M decay rate is almost constant. Similarly, both Asp and Glu residues (Fig. 6, *B* and *C*) also confer constant M decay rates below pH 8. These three donors generally lost their ability at high pH, when the M decay slowed down as the pH increased, as in the ESR-K96Q (Fig. 6*D*). Similar to the case of PR, the mechanism should be different between Lys and carboxyl donors. For the Lys donor, its p*K*a for H^+^ uptake might be located near 7. As a result, M decay exhibited a pH dependence above pH 7. Carboxyl donors were probably deprotonated above pH 8 even in the dark states and thus did not contribute to M decay.

### Hydrostatic pressure dependences of M decay

Donor replacement was not allowed in DR but allowed in PR and ESR. As mentioned previously, we considered that the ESR has a different mechanism than DR and PR. This view might not be the case. For BR, the Asp donor is known to require a CP opening and resultant hydration of the channel, which enables H^+^-transfer reactions mediated by the donor residue (24, 25). This hydration is also likely to occur in PR, reflecting the employment of the same carboxyl donor. However, what about ESR? To probe the magnitude of the hydration, we examined the activation volume associating with the M decay, because the hydration results in the positive activation volume. The volume changes during the photoreactions have been measured for various rhodopsins in different ways including photoacoustic method (26, 27), transient grating method (28, 29), and hydrostatic pressure effects on the kinetics (25, 30). Here, we employed the third method, because the pressure effect can be measured by just introducing the pressure cell into the flash-photolysis apparatus. The hydrostatic pressure slows down the kinetics associated with a positive activation volume, which usually reflects the hydration and/or the expansion of the packing state of the protein (25). For BR, M decay is associated with a large activation volume, which is mainly attributed to the hydration of the CP channel (24, 25).

**Figure 7:**
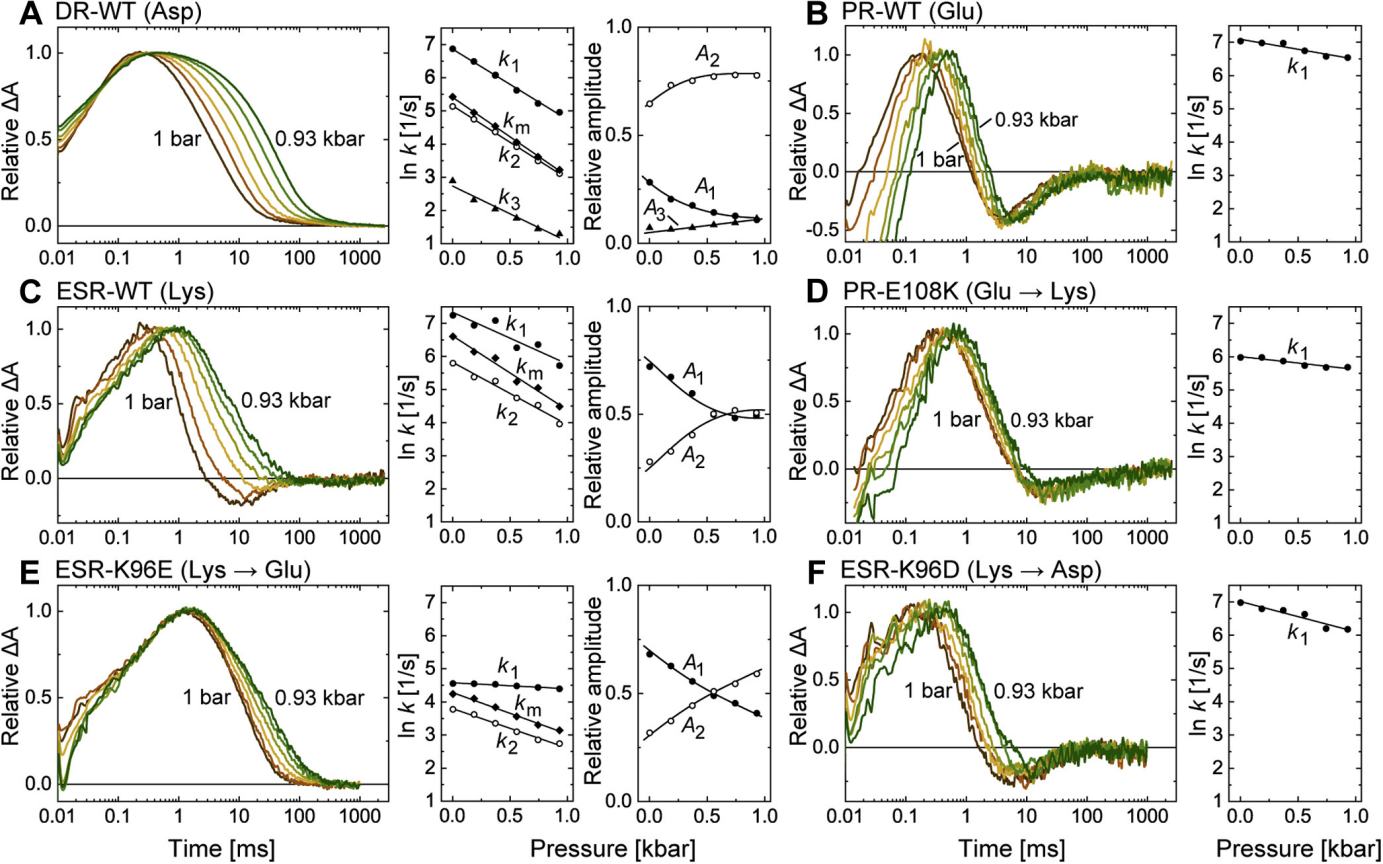
**Hydrostatic pressure dependence of M decay.** The *panels A–C* represent the data for WT DR, PR, and ESR, and the data for donor-replacement mutants of PR (PR-E108K) and ESR (ESR-K96E, ESR-K96D) are shown in *panels D–F*, respectively. Flash-induced absorbance changes (410 nm for DR and ESR; 420 nm for PR) were measured at various hydrostatic pressures and plotted in *larger panels* after normalization with respective peak magnitudes. The pressure values were 1, 190, 370, 560, 740, and 930 bar. All samples were suspended in a salt-free solution at pH 8 to avoid aggregation. In *smaller panels*, the determined fitting parameters for the M decay phases are plotted against the pressure. For details, see the text. DR, deltarhodopsin; ESR, *Exiguobacterium sibiricum* rhodopsin.

Figure 7 shows the flash-induced absorbance changes at 410 or 420 nm under various hydrostatic pressures. The lipid-reconstituted H^+^ pumps tended to aggregate in the presence of the salt. Thus, all data were measured in the absence of NaCl to avoid sample precipitation in the pressure cell, which was not accessible during the measurements. As shown here, the M decay of wildtype DR (Fig. 7*A*) exhibited a significantly large pressure dependence, similar to BR. Large pressure dependence was also observed for wildtype ESR (Fig. 7*C*). Conversely, PR and the mutant exhibited much smaller pressure dependence (Fig. 7, *B* and *D*). The same tendency was also observed for the two ESR mutants (Fig. 7, *E* and *F*). Thus, a smaller pressure dependence might be related to successful donor replacement.

The decay rate constant (*k*) is related to the associated activation volume (Δ*V*^‡^) by Equation 1. Here, we fitted the time traces with multiexponential functions in Equation 2 over a wide time range, including the phase for the formation of M, whose rate constant was denoted by *k*_0_. For the samples except for wildtype DR and ESR-K96E (Fig. 7, *A* and *E*), decay of the next intermediate (N or N-like) was also fitted. Their traces (Fig. 7, *B*–*D* and F) showed negative values before converging to zero. The negative values originated from large accumulations of N or N-like with smaller absorptions than those of the original dark states. Thus, their final increases reflected the decays of N or N-like. The smaller panels in Figure 7 show the determined parameters, the rate constants (*k*_i_) and the amplitude coefficients (*A*_i_), for the phases of M decays and do not include the parameters for other phases (formation of M and decay of N or N-like). A complete set of the parameters is summarized in Fig. S3 together with the fitting curves.

**Figure 8:**
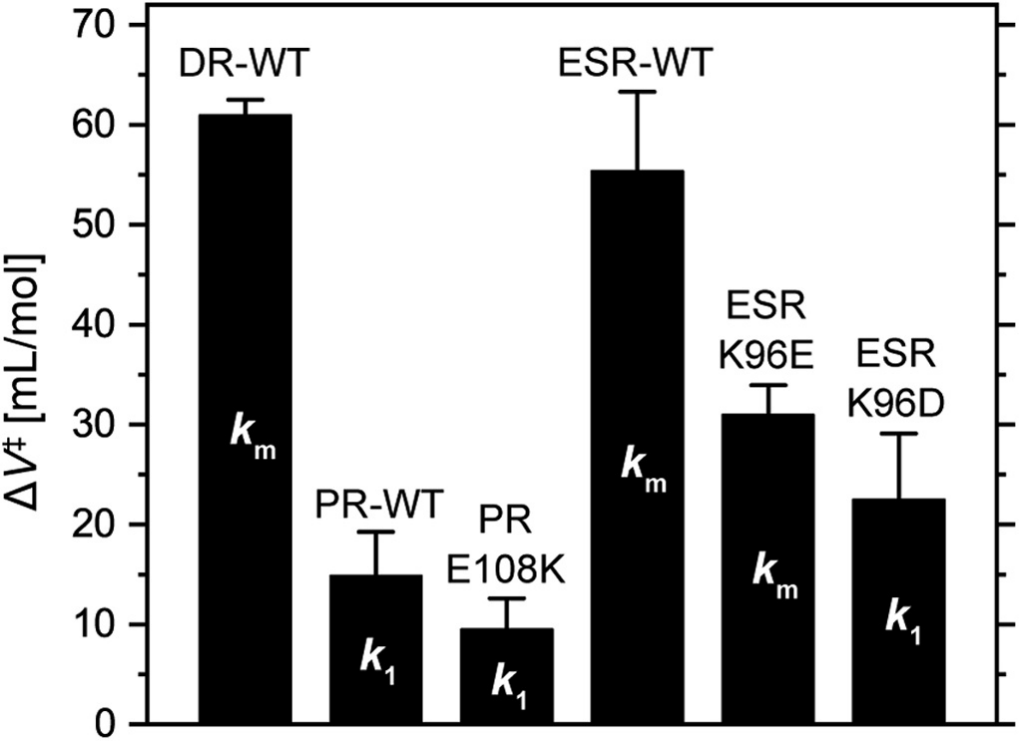
**Activation volumes associated with M decays.** From the pressure dependences of the M decay rates (Fig. 7), the activation volumes (Δ*V*^‡^) were calculated with Equation 1 and plotted together with their standard deviations. For details, see the text.

For wildtype DR (Fig. 7*A*), three exponents (*k*_1_, *k*_2_, and *k*_3_) were required to simulate M decay. Of them, the slowest component (*k*_3_) had a negligible amplitude (*A*_3_). Thus, we focused on the faster components associated with *k*_1_ and *k*_2_. At 1 bar, their corresponding time constants were approximately 1 and 6 ms, respectively. These components also appeared in Figure 5, where the difference in their rate constants became prominent as the pH increased. The first decay rate was not pH dependent and corresponded to *k*_1_, which roughly reflected the formation rate of MN equilibrium. The second decay rate became slower at higher pH and corresponded to *k*_2_, which roughly reflected the decay rate of MN equilibrium. Thus, biexponential decay was caused by the presence of a back reaction from N to M. As a result, both *k*_1_ and *k*_2_ involve complex contributions of rate constants for elementary processes, that is, M → N, N → M, and N → O. As shown later, similar biexponential decays were also observed for wildtype ESR and the K96E mutant. Conversely, for the other three samples (*right panels* in Fig. 7), simple one-step decays were observed. Here, we aimed to compare activation volumes associated with H^+^-transfer reactions by the associated donor residues. Thus, we introduced the “mean rate constant (*k*_*m*_)” (Equation 3) for M decays of DR, ESR, and the K96E mutant. As defined by Equation 3, the *k*_*m*_ is the weighted mean rate of *k*_1_ and *k*_2_, and thus involves two H^+^ transfer processes by the donor, that is, “H^+^ donation” (from donor to SB) and “H^+^ uptake” (from medium to donor). Thus, *k*_m_ roughly reflects the rate for the completion of H^+^ transfers mediated by the donor residue. For DR, the logarithm of the *k*_*m*_ value is plotted in the small panel of Figure 7*A* together with the values for *k*_1_ to *k*_3_. From the slope, the activation volume for *k*_*m*_ was calculated and plotted in Figure 8. This volume was 60.9 ml/mol, which is similar to the reported value for the M to N transition of BR (50.1 ml/mol) (25). This result suggests that the Asp donor in DR requires distinct hydration to mediate H^+^-transfer reactions, similar to BR. The respective activation volumes for *k*_1_ to *k*_3_ were also calculated and summarized in Fig. S4.

For wildtype ESR, the traces also exhibited a large pressure dependence (Fig. 7*C*). The M decay phase at all pressures was fitted well with two decay rates (*k*_1_ and *k*_2_). Until 560 bar, the traces decayed beyond zero and then increased again, reflecting the accumulation of N and the subsequent decay. At these pressures, fourth-rate constants (*k*_3_) were needed to simulate the final increases in the traces. At other pressures, the traces were fitted well with three rate constants (*k*_0_, *k*_1_, and *k*_2_). The first-rate constant (*k*_0_) is associated with the formation of M. Thus, at all pressures, M decays were expressed by two rate constants, *k*_1_ and *k*_2_. For wildtype ESR, M was reported to achieve equilibrium with the subsequent N1 (14). Thus, similar to the case of DR, *k*_1_ roughly associates with the rate for the formation of MN1 equilibrium, whose decay roughly corresponds to *k*_2_. The decay of MN1 equilibrium resulted in the formation of N2 (14), whose accumulation was probably significant until 560 bar and resulted in negative trace values. As shown in the small panel in Figure 7*C*, both *k*_1_ and *k*_2_ exhibited a large pressure dependence. Here, we calculated the mean rate constant *k*_*m*_ by Equation 3 to roughly estimate the activation volume associated with the completion of H^+^ transfers. *k*_*m*_ exhibited a large pressure dependence and was associated with a large activation volume (55.4 ml/mol) comparable to DR (Fig. 8).

In contrast, wildtype PR exhibited very faint pressure dependence (Fig. 7*B*). The traces were only slightly slowed down under high pressure. As mentioned previously, the M decay phases were fitted well with a single decay rate (*k*_1_), whose value also slightly slowed down as the pressure increased (*right panel* in Fig. 7*B*). The lower pressure dependence provided a much smaller activation volume of 14.9 ml/mol compared with BR, DR, and ESR (Fig. 8). For PR, *k*_1_ (M → N transition) reflects the rates of two H^+^ transfers by the donor, that is, “H^+^ donation” and the subsequent “H^+^ uptake” (12). Thus, *k*_1_ for PR roughly corresponds to *k*_*m*_ for DR and ESR. Nevertheless, the donor in PR only needs very low levels of hydration for the H^+^-transfer reactions. Faint pressure dependence was also observed for the donor replacement mutant PR-E108K (Fig. 7*D*). The M decay of PR-E108K was also fitted well with a single decay rate, which involved two H^+^-transfer reactions, as for the wildtype PR, but their order was inverted because “H^+^ uptake” should occur first, followed by “H^+^ donation”. The faint pressure dependence resulted in a smaller activation volume of 9.5 ml/mol (Fig. 8), suggesting that neither H^+^ transfer requires large hydrations.

Significantly smaller pressure dependences were also observed for the two ESR mutants. For K96E (Fig. 7*E*), the M decays were fitted well with two decay rates (*k*_1_ and *k*_2_), probably reflecting the formation of MN equilibrium and its subsequent decay. Similar to DR and ESR, we calculated *k*_*m*_. The determined values showed a smaller pressure dependence compared with the wildtype ESR and thus led to a smaller activation volume of 31.0 ml/mol. For K96D (Fig. 7*F*), the M decays were well fitted with a single decay rate, whose pressure dependence was very small and led to a smaller activation volume of 22.5 ml/mol. It is not clear whether this M decay also involves H^+^ uptake, similar to wildtype PR. Even if this is not the case, the H^+^ capture process should also require a faint activation volume. H^+^ capture should occur during the decay of subsequent N- or N-like intermediate, which corresponds to the final increase in the traces. These traces did not show any pressure dependence.

## Discussion

### Donor replaceabilities among three H^+^ pumps

The excellent H^+^ donor residue should confer a pH-independent “fast” M decay at least around physiological pH. The donor-replacement mutants of PR and ESR indeed exhibited the pH-independent M decays in respective pH ranges (Figs. 5 and 6). Thus, donor replacements were surely allowed in PR and ESR. In contrast, DR-D104K exhibited significant pH dependence in the M decay at all measured pH values (Fig. 5*B*). Thus, Lys residue cannot act as H^+^ donor in DR. As mentioned previously, we initially expected that the donor replacements might not be allowed between carboxyl-type and Lys-type H^+^ pumps because of their optimized conformational changes for respective H^+^ donor residues. This view seems to be consistent with the results for DR-D104K. The Asp donor in DR should be first allowed access to the SB, but Lys donor needs to first access the CP medium to capture H^+^. Thus, conformational change of DR might disturb the H^+^-transfer reactions of Lys residue. However, this “mismatch” might be covered by the distorted protein structure. As shown in Figures 2 and 5, DR-D104K exhibited significantly faster M decay compared with DR-D104N. This result probably originated from the steric effect. The embedded Lys residue is likely to distort the protein structure and then facilitate H^+^ inflow from the medium. The faint expression level of DR-D104K (Fig. 4*C*) might also reflect the steric effect. The CP channel of DR might be too tight to accommodate the Lys residue, so that its introduction might disturb structural formation in the cell membrane. Thus, “true” replaceability of the donor residue probably cannot be discussed for DR.

As shown in Figure 4*B*, PR-E108K exhibited significantly smaller H^+^-pump activity in spite of the fast M decay comparable to the wildtype PR (Fig. 2). This weak activity probably reflected the slow photocycling rate because of the prolonged N decay. For PR-E108K, the H^+^ transfers at CP side should be completed until the M decay. Thus, the subsequent N decay does not appear to be affected by the donor replacement. However, the replacement might distort the protein conformation, which might slow down the processes involved in the N decay, such as reisomerization of retinal. The PR-E108K exhibited a 13-nm redshift of the absorption spectrum in the dark state (Fig. S2). This redshift might originate from the distorted protein conformation.

### A possible difference between archaeal and eubacterial H^+^ pumps

**Figure 9:**
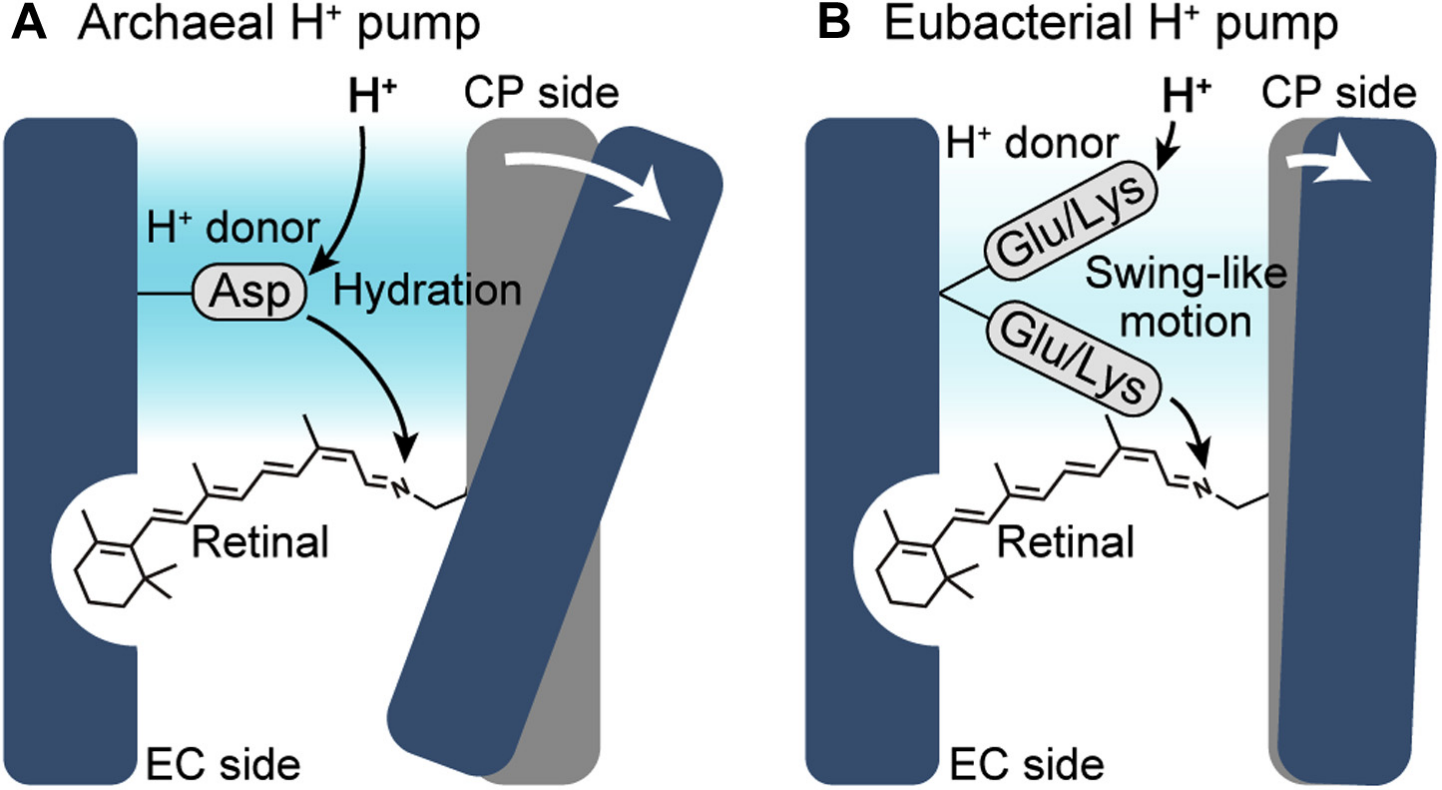
**The model of the H**^**+**^**-transfer reactions of archaeal and eubacterial H**^**+**^**pumps.***A*, archaeal BR and DR cause large CP opening and significant hydration, which are prerequisite for the H^+^-transfer reactions. *B*, eubacterial PR and ESR perform the H^+^-transfer reactions in the reverse order, but they share the mechanism, which utilizes the swing-like motion of donor residue instead of the hydration. BR, bacteriorhodopsin; CP, cytoplasmic; DR, deltarhodopsin; ESR, *Exiguobacterium sibiricum* rhodopsin; PR, proteorhodopsin.

We believed that DR and PR commonly utilize the hydration for the H^+^-transfer reactions, as revealed for BR. As expected, the H^+^ transfers of DR are associated with a large activation volume, which probably reflects significant hydration of the CP channel. Thus, BR and DR share the H^+^ transfer mechanism, which is schematically shown in Figure 9*A*. Conversely, the H^+^ transfers of PR require much smaller activation volumes. This feature was conserved even in the E108K mutant. Thus, PR and the mutant seem to generally require lower levels of hydration of the channels. However, the CP channel of PR is also hydrophobic, similar to BR and DR. Why is lower hydration allowed for PR? One possibility might be the high flexibility of the donor residues as illustrated in Figure 9*B*. If the CP channel of PR allows a swing-like motion of the donors, less hydration is probably sufficient for the donors to mediate the H^+^-transfer reactions. Thus, Glu and Lys donors in PR might share a mechanism, which builds on the swing-like motion of the donors but not on the hydration of the channel. This common mechanism might allow donor replacement in PR. The Lys introduction into DR probably disturbed the protein structure. Thus, archaeal H^+^ pump might have lower flexibility of CP channel and inhibit the swing-like motion of its donor residue. Consequently, archaeal DR and BR might require much hydration of the channel to facilitate the H^+^-transfer reactions (Fig. 9*A*).

Is high flexibility also true for the wildtype ESR and the mutants? Smaller activation volumes for two mutants (ESR-K96E and ESR-K96D) suggest a common mechanism with PR, that is, the embedded Glu and Asp donors seem to exert a swing-like motion and facilitate the H^+^-transfer reactions without significant hydration. Thus, ESR seems to share flexibility with PR. Indeed, previous X-ray structural analysis of ESR revealed some extent mobility of the donor residue, where the Lys side chains took different conformations in the antiparallel ESR dimers with different electron densities (31). Based on these observations, a flexible motion of the donor was suggested to have a potential role for the H^+^-transfer reactions. Compared with the mutants, we observed a large activation volume for the wildtype ESR, similar to BR and DR. However, ESR and archaeal H^+^ pumps undergo significantly different H^+^-transfer reactions. Thus, the large activation volume of ESR might also reflect a different conformational change from archaeal H^+^ pumps. This puzzling situation might be explained by the moderate flexibility of ESR compared with PR. Even with this flexibility, ESR probably allows the swing-like motion of the embedded Glu and Asp donors without significant conformational change. However, for the motion of the original Lys donor, a large expansion of the packing state of the protein might be necessary during M decay. Similar to the discussion for PR (Fig. 9*B*), we assumed that the swing-like motion of the donor is a key mechanism not only for the wildtype ESR but also for the donor-replacement mutants. This common mechanism might be the reason that successful donor replacements were achieved.

In ESR, the embedded Asp residue can act as an excellent H^+^ donor, similar to Glu and original Lys residues. However, the Asp side chain is shorter than the other side chains. Thus, for Asp residue, the swing-like motion might not be enough to facilitate the H^+^ relay reactions. This disadvantage might be overcome by the displacements of the water molecules inside the protein. The Lys-to-Asp replacement is likely to create the cavity, which is probably filled by the water molecules in the dark state. In the photolyzed state, these water molecules might move together with the Asp side chain to assist the H^+^-transfer reactions. This mechanism does not involve further hydration and the resulting volume increase.

Unlike archaeal H^+^ pumps, PR and ESR appeared to cause smaller hydration of the CP channel. Especially, PR and its E108K mutant exhibited much smaller activation volumes, indicating that only slight hydration occurred during the M decay. Different amounts of hydration should originate from different conformational changes. In the photolyzed BR, the CP portion of helix F causes outward tilting and then induces the hydration of the CP channel. The initial key events for this conformational change are considered to be the displacement of the 13-methyl group of the retinal and the twist of the Lys216 side chain, which connects the retinal with helix G *via* SB linkage (for reviews, see Refs. (11, 32)). Even in other H^+^ pumps, similar conformational changes had been considered to occur with similar mechanism. However, this view might not be the case at least for PR and ESR. These H^+^ pumps appear to cause smaller conformation changes. Moreover, the positions of these conformational changes can also differ from BR. Thus, the comparisons of conformational changes and the resultant donor flexibilities among H^+^ pumps should be interesting subjects in the future investigations.

## Conclusion

BR is the best studied microbial rhodopsin, and the details of the H^+^ pumping mechanism have been vigorously analyzed. Concerning the donor residue, its H^+^-transfer reactions were revealed to require a large amount of hydration of the CP channel (24, 25). This hydration also was supposed to be important for other H^+^ pumps because all H^+^ pumps commonly involve hydrophobic CP channels. The present study indeed confirmed the large hydration for archaeal DR. However, eubacterial H^+^ pumps of PR and ESR were suggested to adopt a different mechanism, in which the donor residues seem to require only smaller hydrations. To mediate the H^+^-transfer reactions, these donors might swing the side chains in the CP channels. This common mechanism potentially allows donor replacements in PR and ESR. For DR, the embedded Lys residue probably distorted the conformation of the CP channel. This result seems to reflect the lower flexibility of the CP channel, which might also inhibit the swing-like motion of the original donor residue even in the photolyzed state. As a result, the donors in DR and BR might require hydration of the CP channels. Despite the differences in the donor residues, PR and ESR were phylogenetically close (Fig. S1) compared with DR and BR. Thus, these eubacterial H^+^ pumps might possess a common mechanism that is distinct from the mechanism in archaeal H^+^ pumps.

## Experimental procedures

### Protein expression, purification, and lipid reconstitution

*E. coli* DH5α was used for DNA manipulation. The expression plasmids of DR and PR were described previously (17, 23). They were encoded in pET-21c and pBAD vectors, respectively. The ESR gene (GenBank accession no.: ACB60885) with codons optimized for *E. coli* expression was chemically synthesized (Funakoshi) and inserted into the pKA001 vector under the lacUV5 promoter with NdeI and HindIII sites (33, 34). All plasmids resulted in proteins having a six-histidine tag in the C terminus. Mutations of donor residues were introduced using the QuikChange Site-Directed Mutagenesis Kit (Agilent Technologies). The DNA sequences were confirmed by a standard procedure.

For expression and purification, *E. coli* strains BL21(DE3), UT5600, and BL21 were used for the wildtype and mutant DR, PR, and ESR, respectively. The respective procedures were essentially the same as those previously described (23, 35, 36). Briefly, the cells were grown in 2× YT medium at 37 °C, and expression was induced in the presence of 10 μM all-*trans* retinal by the addition of 1 mM isopropyl-β-D-thiogalactopyranoside for DR and ESR or 0.2% l-(+)-arabinose for PR. After 3 to 4 h of induction, the cells were harvested and broken with a French press. The collected cell membrane fractions were solubilized with 1.5% *n*-dodecyl β-D-maltopyranoside, and the solubilized proteins were purified using nickel–nitrilotriacetic acid agarose. The concentrations of the proteins were determined from the absorbances at λ_max_ values of approximately 520 to 540 nm by assuming extinction coefficients of 63,000 M^−1^ cm^−1^ for DR and 50,000 M^−1^ cm^−1^ for PR and ESR. The purified samples were replaced with an appropriate buffer solution by passage over Sephadex G-25 in a PD-10 column (Amersham Bioscience).

Lipid reconstitution was performed as previously described (37). For reconstitution into the lipid, phosphatidylcholine from egg yolk (Avanti) was added at a protein: phosphatidylcholine molar ratio of 1:50. The detergent was removed by gentle stirring overnight (4 °C) in the presence of SM2 Adsorbent Bio-Beads (Bio-Rad). After filtration, the reconstituted proteins were collected by centrifugation, and the buffer solutions were replaced with 6-mix buffer (citric acid, 1.37 mM; ADA, 0.46 mM; MOPS, 1.04 mM; TAPS, 1.25 mM; CHES, 0.81 mM; and CAPS, 1.39 mM) with or without 0.4 M NaCl.

### Flash photolysis spectroscopy

The flash photolysis apparatus equipped with a neodymium-doped yttrium aluminium garnet laser (532 nm, 7 ns) was described previously (38, 39). The suspensions of the reconstituted proteins were illuminated by laser pulses. The subsequent absorbance changes were recorded with a single wavelength kinetic system. To improve the S/N ratio, 30 laser pulses were used at each measurement wavelength. The protein concentrations were approximately 5 to 10 μM, and the temperature was maintained at 25 °C. Data points on a logarithmic time scale were picked up from the observed data and used for plots and the fitting analysis. For pH titration experiments, the pH values were adjusted by adding a small amount of H_2_SO_4_ or NaOH.

Flash photolysis under various pressures was measured by replacing the sample holder with a pressure cell, whose details were described previously (30). The cylindrical sample cuvette (5 mm optical path) was sealed with PARAFILM (American National Can) and set inside the pressure cell filled with water. The pressure was adjusted between 1 and 930 bar by a hand-operated hydraulic pump using water as a pressurizing medium. The lipid-reconstituted proteins tended to gradually aggregate in the presence of NaCl. Thus, the samples for this experiment were suspended in 6-mix buffer, pH 8, that did not contain NaCl.

The decay rate constant (*k*) of the intermediate has the following relationship with the associated activation volume (Δ*V*^‡^):

(1) $$\ln\;k=\text{constant }-\frac{P\Delta V^{‡}}{R\;T}$$

where *P*, *R*, and *T* denote the hydrostatic pressure, gas constant, and absolute temperature, respectively. To determine the rate constant, we fitted the time traces of absorption change (Δ*A*(*t*)) with the following multiexponential functions:

(2) $$\text{Δ}A\left(t\right)=\;\overset{n}{\underset{i=0}{\sum }}A_{i}\;\text{exp}\left(-k_{i}\;t\right)$$

where *A*_*i*_ and *k*_*i*_ denote the amplitude coefficient and decay rate constant, respectively. The M decays of DR, ESR, and the ESR-K96E were expressed by two rate constants, *k*_1_ and *k*_2_. For these H^+^ pumps, the following “mean rate constant (*k*_*m*_)” was introduced:

(3) $$k_{m}=\;\frac{1}{\tau_{m}}=\frac{A_{1}+A_{2}}{A_{1}\tau_{1}+A_{2}\tau_{2}}=\frac{A_{1}+A_{2}}{A_{1}/k_{1}+A_{2}/k_{2}}$$

where *τ*_*m*_, *τ*_1_, and *τ*_2_ are the time constants and reciprocals of *k*_*m*_, *k*_1_, and *k*_2_, respectively.

### Proton pump activity measurements

The H^+^ pumping activities of the wildtype and mutant proteins were measured in *E. coli* suspensions using a conventional pH electrode method. The experimental details were almost the same as those previously reported (40). Briefly, the cells expressing the H^+^ pump rhodopsin were harvested and washed twice with a salt solution containing 200 mM NaCl and 10 mM MgCl_2_. The cell suspensions were gently shaken overnight and then washed twice in the same solution. Finally, the cells were suspended in a 10 ml volume at an absorbance of 2.0 at 660 nm. The suspensions were illuminated by green LED light of 530 ± 17.5 nm (LXHL-LM5C; Philips Lumileds Lighting Co). The light intensity was adjusted to approximately 24 mW/cm^2^ at 530 nm using an optical power meter (Orion-PD; Ophir Optronics Ltd). All measurements were performed at room temperature (approximately 25 °C).

The H^+^-pump activities were evaluated by the initial slopes of pH decreases upon illumination. However, these slopes depend on the expression levels of the H^+^ pump rhodopsins. The expression levels were estimated by the magnitudes of the flash-induced absorbance changes. The procedures were previously described (40). After the measurement of H^+^-pump activities, the cells were collected by centrifugation and then suspended in 2 ml of 50 mM Mes, pH 6, and 200 mM NaCl. After disrupting the cells by sonication, the lysates were used for the measurements of flash-induced absorbance changes. The measurement wavelengths were set to the λ_max_ values of the absorption spectra in the *n*-dodecyl β-D-maltopyranoside–solubilized state. The negative deflected signals reached maximum values within 0.1 to 10 ms. These values were employed as the relative expression amounts of the H^+^ pumps. To calculate the relative H^+^-pump activities, the initial slopes of pH decreases were divided by the maximum values of the flash-induced absorbance changes.

## Data availability

All the data supporting the findings of this study are available within the article and supporting information.

## Supporting information

This article contains supporting information.

## Conflict of interest

The authors declare that they have no conflicts of interest with the contents of this article.
