# Supplementary material for: Replaceability of Schiff base proton donors in light-driven proton pump rhodopsins
Source: J Biol Chem. 2021 Jul 28;297(3):101013. doi: 10.1016/j.jbc.2021.101013 (PMC8387761; doi:10.1016/j.jbc.2021.101013)
Supplement: Supplemental Figures S1–S4 [file mmc1.pdf]

Supporting Information

Replaceability of Schiff base proton donors in light-driven proton pump rhodopsins

Syogo Sasaki,[#,†] Jun Tamogami,[*,#,§] Koki Nishiya,[†] Makoto Demura,[‡] Takashi Kikukawa[*,‡]

[†] Graduate School of Life Science, Hokkaido University, Sapporo 060-0810, Japan

[§] College of Pharmaceutical Sciences, Matsuyama University, Matsuyama, Ehime 790-8578, Japan

[‡] Faculty of Advanced Life Science, Hokkaido University, Sapporo 060-0810, Japan

[#] These authors contributed equally to this work.

*Corresponding author

Dr. Jun Tamogami, E-mail: jtamoga@g.matsuyama-u.ac.jp.

Dr. Takashi Kikukawa, E-mail: kikukawa@sci.hokudai.ac.jp.

Table of Contents

Supplemental Figures S3

Figure S1. Phylogenetic relationships among bacterial rhodopsins.. S3

Figure S2. Absorption spectra of the samples used in this study.. S4

Figure S3. Fitting analysis results of the flash-induced absorbance changes

at various hydrostatic pressures... S5

Figure S4. Activation volumes associated with M decays.. S6

Supplemental Figures

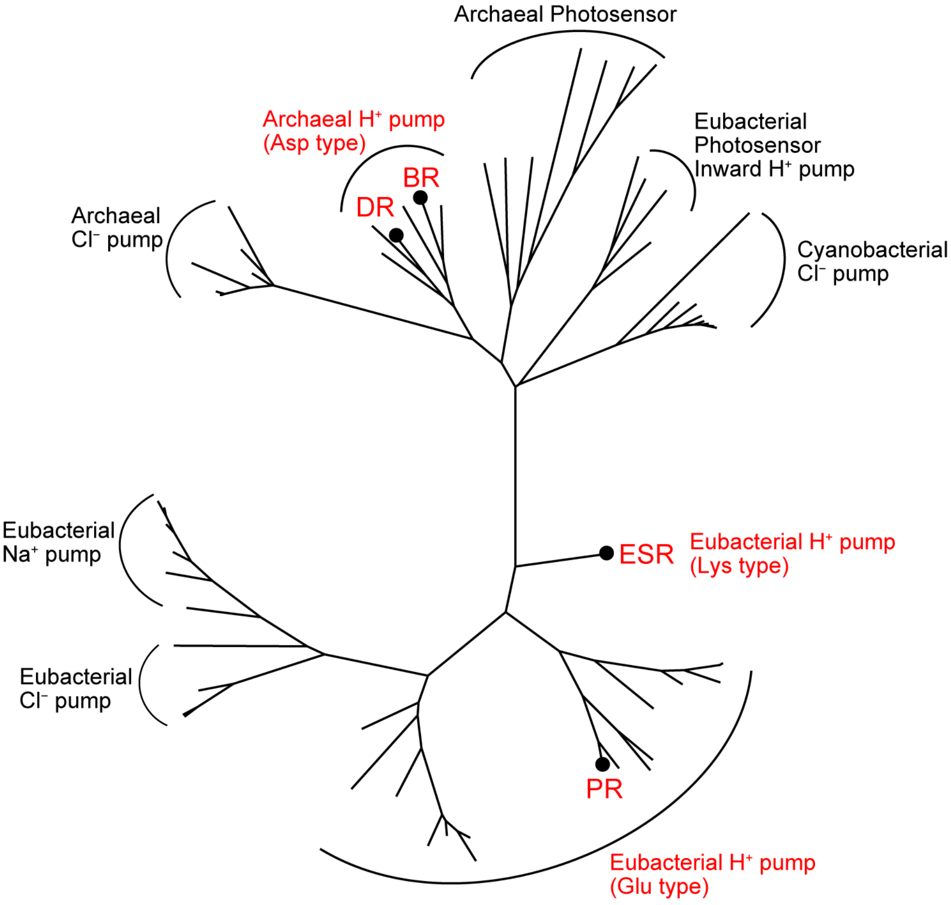

Figure S1. Phylogenetic relationships among bacterial rhodopsins

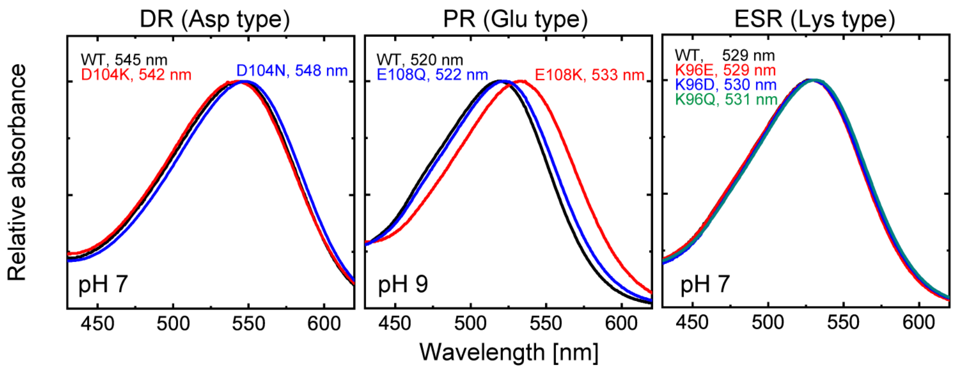

Figure S2. Absorption spectra of the samples used in this study. The respective λ_{max} are indicated in the panels. All spectra were measured in the presence of 0.3 M NaCl and 0.1% DDM. The pH values were 7 for DR and ESR and 9 for PR.

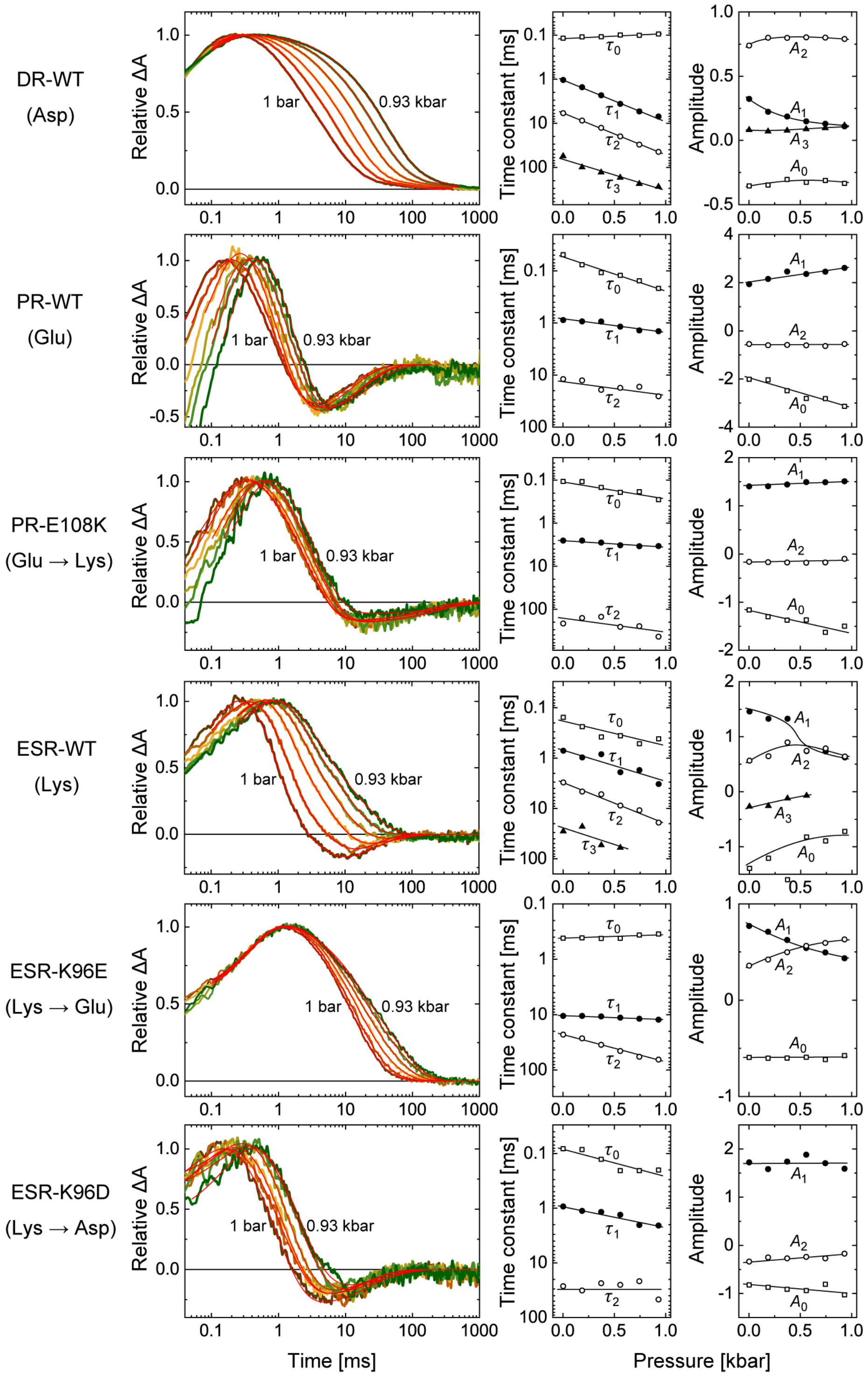

Figure S3. Fitting analysis results of the flash-induced absorbance changes at various hydrostatic pressures. The detailed version of Fig. 7 is shown here. In large panels, the best-fitted curves are shown with red smooth lines. Instead of the rate constants (k_i) in Fig. 7, the corresponding time constants ($\tau_i = 1/k_i$) are plotted in the middle panels. For all samples, τ_0 and A_0 represent the phases of M formations.

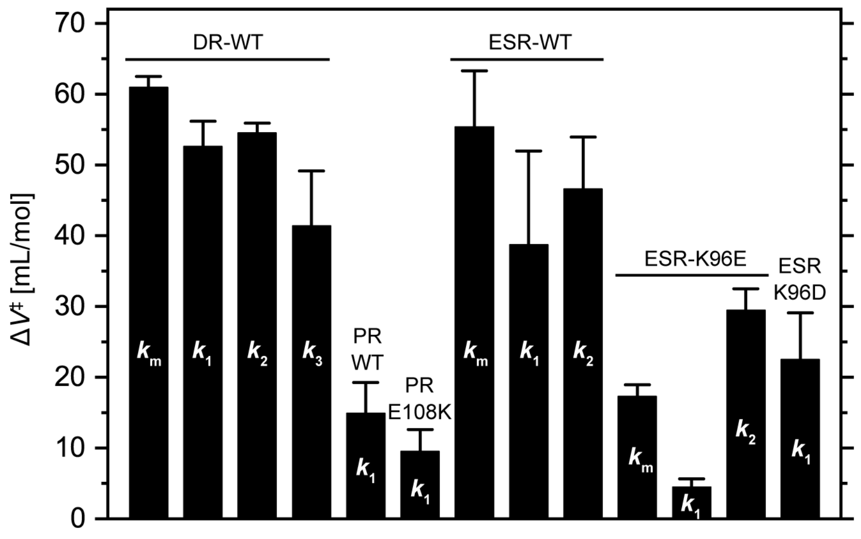

Figure S4. Activation volumes associated with M decays. The detailed version of Fig. 8 is shown here. Error bars indicate the standard deviations. In addition to the activation volumes for k_m, those values for individual rate constants are shown for wild-type DR, wild-type ESR and its K96E mutant.
